# Prognosis Analysis and Validation of m^6^A Signature and Tumor Immune Microenvironment in Glioma

**DOI:** 10.3389/fonc.2020.541401

**Published:** 2020-10-05

**Authors:** Shaojian Lin, Houshi Xu, Anke Zhang, Yunjia Ni, Yuanzhi Xu, Tong Meng, Mingjie Wang, Meiqing Lou

**Affiliations:** ^1^Department of Neurosurgery, Shanghai General Hospital, Shanghai Jiao Tong University School of Medicine, Shanghai, China; ^2^School of Medicine, Tongji University, Shanghai, China; ^3^Department of Orthopedics, Shanghai General Hospital, Shanghai Jiao Tong University School of Medicine, Shanghai, China; ^4^Department of Digestive Diseases, Ruijin Hospital North, Shanghai Jiao Tong University School of Medicine, Shanghai, China

**Keywords:** glioma, m^6^A, immune infiltration, WGCNA, prognostic model

## Abstract

Glioma is one of the most typical intracranial tumors, comprising about 80% of all brain malignancies. Several key molecular signatures have emerged as prognostic biomarkers, which indicate room for improvement in the current approach to glioma classification. In order to construct a more veracious prediction model and identify the potential prognosis-biomarker, we explore the differential expressed m^6^A RNA methylation regulators in 665 gliomas from TCGA-GBM and TCGA-LGG. Consensus clustering was applied to the m6A RNA methylation regulators, and two glioma subgroups were identified with a poorer prognosis and a higher grade of WHO classification in cluster 1. The further chi-squared test indicated that the immune infiltration was significantly enriched in cluster 1, indicating a close relation between m^6^A regulators and immune infiltration. In order to explore the potential biomarkers, the weighted gene co-expression network analysis (WGCNA), along with Least absolute shrinkage and selection operator (LASSO), between high/low immune infiltration and m^6^A cluster 1/2 groups were utilized for the hub genes, and four genes (*TAGLN2, PDPN, TIMP1, EMP3)* were identified as prognostic biomarkers. Besides, a prognostic model was constructed based on the four genes with a good prediction and applicability for the overall survival (OS) of glioma patients (the area under the curve of ROC achieved 0.80 (0.76–0.83) and 0.72 (0.68–0.76) in TCGA and Chinese Glioma Genome Atlas (CGGA), respectively). Moreover, we also found *PDPN* and *TIMP1* were highly expressed in high-grade glioma from The Human Protein Atlas database and both of them were correlated with m6A and immune cell marker in glioma tissue samples. In conclusion, we construct a novel prognostic model which provides new insights into glioma prognosis. The *PDPN* and *TIMP1* may serve as potential biomarkers for prognosis of glioma.

## Introduction

Glioma is a common primary tumor in the central nervous system (CNS), accounting for about 80% of brain malignancies (1, 2). The lower-grade gliomas (LGGs) has a relatively favorable prognosis, consisting of the diffuse low-grade and intermediate-grade gliomas (World Health Organization [WHO] grades II and III), whereas glioblastoma (GBM) are generally high-grade gliomas (grade IV) (3, 4). Despite recent medical advances, patients with high-grade GBM are still associated with poor prognosis. Thus, identifying the difference in various gliomas may assist oncologists in finding the prognostic biomarkers and potential targets for glioma patients.

N6-Methyladenosine (m^6^A) is the most popular internal mRNA modification in diverse cell types and consists of the m6A methyltransferases, reverted by the demethylases and identified by m^6^A binding proteins (5–10). Generally, m^6^A modification has various regulatory functions in tumorigenesis, progression and immunity modulation (11–15). Meanwhile, tumor immune microenvironment also participates in tumor initiation and progression and influences the clinical outcomes of patients (16–18). Immune classification of cancers is crucial in therapeutic strategy establishing and prognosis assessment of patients with tumors (19, 20).

Several studies have revealed the correlation between tumor microenvironment (TME) infiltrating immune cells and m^6^A modification. In the gastric tumors, m6A modification patterns could predict the stages of tumor inflammation, TME stromal activity, genetic variation and patient prognosis. Lower m6A score indicated an inflamed TME phenotype and enhanced response to anti-PD-1/L1 immunotherapy (21). The high expression of WTAP, a m6A methyltransferase, was also associated with RNA methylation and its low expression was related to a high T cell-related immune response in gastric cancer (22). Additionally, m^6^A was reduced in the high immunity subtype of lung adenocarcinoma, indicating that m^6^A may mediate immune signatures and help to provide potential strategies (23). However, the potential roles of m6A modification in immune infiltration remain obscure, especially in glioma. Therefore, identification of immune infiltration characterizations mediated by multiple m6A regulators might be helpful for the survival prognosis of patients with glioma.

In this study, in order to investigate the novel prediction model and potential biomarkers for glioma, WGCNA and LASSO were applied to identify candidate genes that might take part in both m6A and immune infiltration in glioma based on TCGA database. Differentially expressed genes (DEGs) were identified, along with their prognostic values, and further validated by external datasets and tissue microarray. Besides, the constructed prediction model revealed a high efficacy for prognosis prediction. The potential predictive biomarkers were also identified to assist oncologists in clinic treatment.

## Methods and Materials

### Datasets Acquisition From TCGA Datasets

The Cancer Genome Altas (TCGA) GBMLGG datasets (*n* = 665) were downloaded from the University of California Santa Cruz (UCSC) Xena browser (https://xenabrowser.net/datapages/). The gene expression data were presented as FPKM values derived from TCGA level 3 data. Batch effects were removed before analyzing (24). Clinical data of TCGA datasets were downloaded from the UCSC Xena browser, including clinical information (age, gender), tumor information (subtypes) and survival information (overall survival) for patients with gliomas (Table 1). The RNA-seq transcriptome data and corresponding clinicopathological information of 420 LGG patients and 237 GBM patients were obtained from CGGA (www.cgga.org.cn) as a validation set. The RNA-seq transcriptome data were transformed as FPKM values. GSE16011 (25) expression data was downloaded from GEO database. Robust multi-array average (RMA) normalized files were used in this study. The probe was converted into gene symbol by median gene expression. The microarray data were estimated as log2(x+1) normalized expression value.

**Table 1:** Summary table of the TCGA clinical information.

	**Level**	**Cluster1**	**Cluster2**	***p***
*N*		190	475	
Study (%)	GBM	87 (46.8)	63 (13.4)	<0.001
	LGG	99 (53.2)	407 (86.6)	
Grade (%)	II	42 (23.9)	182 (41.8)	<0.001
	III	48 (27.3)	191 (43.9)	
	IV	87 (49.1)	63 (14.4)	
Histology (%)	Astrocytoma	42 (22.6)	149 (31.7)	<0.001
	GBM	87 (46.8)	63 (13.4)	
	Oligoastrocytoma	28 (15.1)	100 (21.3)	
	Oligodendroglioma	29 (15.6)	158 (33.6)	
Recurrence (%)	Primary	176 (92.6)	432 (90.9)	NA
Subtype (%)	Classic-like	33 (20.0)	34 (7.5)	<0.001
	Codel	26 (15.8)	143 (31.6)	
	G-CIMP-high	54 (32.7)	178 (39.4)	
	G-CIMP-low	8 (4.8)	7 (1.5)	
	LGm6-GBM	4 (2.4)	6 (1.3)	
	Mesenchymal-like	36 (21.8)	62 (13.7)	
	PA-like	4 (2.4)	22 (4.9)	
survival [mean (*SD*)]		25.96 (31.55)	27.31 (28.24)	0.596
status [mean (*SD*)]		0.50 (0.50)	0.31 (0.46)	<0.001
Transcriptome.Subtype (%)	CL	36 (23.4)	48 (13.1)	<0.001
	ME	38 (24.7)	57 (15.5)	
	NE	13 (8.4)	96 (26.2)	
	PN	67 (43.5)	166 (45.2)	
Pan_Glioma.RNA.Expression.Cluster (%)	LGr1	36 (19.5)	102 (21.8)	<0.001
	LGr2	11 (5.9)	77 (16.5)	
	LGr3	59 (31.9)	174 (37.2)	
	LGr4	79 (42.7)	115 (24.6)	
IDH_specific.RNA.Expression.Cluster (%)	IDHmut-R1	15 (8.2)	89 (19.2)	<0.001
	IDHmut-R2	14 (7.7)	82 (17.7)	
	IDHmut-R3	59 (32.2)	157 (33.9)	
	IDHwt-R1	22 (12.0)	24 (5.2)	
	IDHwt-R2	34 (18.6)	44 (9.5)	
	IDHwt-R3	30 (16.4)	38 (8.2)	
	IDHwt-R4	9 (4.9)	29 (6.3)	
Pan_Glioma.DNA.Methylation.Cluster (%)	LGm1	19 (11.4)	30 (6.6)	<0.001
	LGm2	51 (30.5)	199 (43.4)	
	LGm3	19 (11.4)	102 (22.3)	
	LGm4	32 (19.2)	32 (7.0)	
	LGm5	37 (22.2)	67 (14.6)	
	LGm6	9 (5.4)	28 (6.1)	
IDH_specific.DNA.Methylation.Cluster (%)	IDHmut-K1	16 (9.7)	24 (5.3)	<0.001
	IDHmut-K2	46 (27.9)	162 (35.8)	
	IDHmut-K3	26 (15.8)	143 (31.6)	
	IDHwt-K1	33 (20.0)	34 (7.5)	
	IDHwt-K2	36 (21.8)	62 (13.7)	
	IDHwt-K3	8 (4.8)	28 (6.2)	
Subtype.original (%)	Classical	25 (13.5)	13 (2.8)	<0.001
	G-CIMP	6 (3.2)	2 (0.4)	
	IDHmut-codel	25 (13.5)	140 (30.0)	
	IDHmut-non-codel	57 (30.8)	186 (39.8)	
	IDHwt	16 (8.6)	79 (16.9)	
	Mesenchymal	26 (14.1)	22 (4.7)	
	Neural	11 (5.9)	15 (3.2)	
	Proneural	19 (10.3)	10 (2.1)	
Random.Forest.Sturm.Cluster (%)	G34	0 (0.0)	1 (0.2)	0.175
	IDH	81 (65.3)	318 (74.5)	
	K27	0 (0.0)	1 (0.2)	
	Mesenchymal	22 (17.7)	67 (15.7)	
	RTK I ‘PDGFRA’	3 (2.4)	9 (2.1)	
	RTK II ‘Classic’	18 (14.5)	31 (7.3)	
IDH.status (%)	Mutant	89 (48.4)	330 (71.0)	<0.001
	WT	95 (51.6)	135 (29.0)	
Chr.1p_19q.codeletion (%)	Codel	25 (13.7)	140 (29.9)	<0.001
	non-codel	158 (86.3)	328 (70.1)	
IDH_codel.subtype (%)	IDHmut-codel	25 (13.8)	140 (30.2)	<0.001
	IDHmut-non-codel	64 (35.4)	188 (40.6)	
	IDHwt	92 (50.8)	135 (29.2)	
MGMT.promoter.status (%)	Methylated	117 (70.1)	353 (77.1)	0.091
	Unmethylated	50 (29.9)	105 (22.9)	
Chr.7.gain_Chr.10.loss (%)	Gain chr 7 & loss chr 10	69 (37.9)	81 (17.4)	<0.001
	No combined can	113 (62.1)	385 (82.6)	
Chr.19_20.co_gain (%)	Gain chr 19/20	12 (6.6)	18 (3.9)	0.201
	No chr 19/20 gain	170 (93.4)	448 (96.1)	
TERT.promoter.status (%)	Mutant	39 (47.0)	113 (49.1)	0.836
	WT	44 (53.0)	117 (50.9)	
TERT.expression.status (%)	Expressed	118 (63.8)	227 (48.5)	0.001
	Not expressed	67 (36.2)	241 (51.5)	
ATRX.status (%)	Mutant	46 (25.1)	146 (31.5)	0.132
	WT	137 (74.9)	317 (68.5)	
DAXX.status (%)	Mutant	2 (1.1)	0 (0.0)	0.142
	WT	181 (98.9)	463 (100.0)	
Telomere.Maintenance (%)	-/-	15 (18.1)	35 (15.5)	0.86
	ATRX	29 (34.9)	82 (36.3)	
	TERT	39 (47.0)	109 (48.2)	
BRAF.V600E.status (%)	Mutant	1 (0.5)	2 (0.4)	1
	WT	182 (99.5)	461 (99.6)	
BRAF_KIAA1549.fusion (%)	Fusion	0 (0.0)	1 (0.2)	1
	WT	185 (100.0)	467 (99.8)	
RPPA.cluster (%)	K1	47 (49.5)	46 (20.5)	<0.001
	K2	48 (50.5)	178 (79.5)	

### Selection of m^6^A RNA Methylation Regulators

We used 12 m^6^A RNA methylation regulators from published literature. Then, the expression of these m6A RNA methylation regulators in gliomas were systematically compared with different clinical outcomes using Gliovis (http://gliovis.bioinfo.cnio.es/) (26).

### Unsupervised Analysis With ConsensusClusterPlus

In order to investigate the function of m^6^A RNA methylation regulators in glioma, we divided patients with glioma into different groups with “ConsensusClusterPlus” (50 iterations, resample rate of 80%). The principal component analysis was then performed with the R package “PCA” for R v3.5.1 to study the gene expression patterns in different glioma clusters. In order to determine the optimal K, Average Silhouette method and Gap Statistic method were applied, the results showed that the two groups were the best grouping number (Supplementary Figure 1). Wilcoxon signed rank test was used to compare the tumor mutation burden of cluster 1 and cluster 2.

### Function Analysis of m^6^A Cluster Subgroups and Immune Infiltration Analysis Based on Single-Sample Gene Set Enrichment Analysis (ssGSEA)

Gene Set Variation Analysis (GSVA) was performed with the R package “gsva” to evaluate pathway enrichment for different clusters. To investigate the immune infiltration landscape of glioma, ssGSEA was performed to assess the level of immune infiltration (recorded as ssGSEA score) in a sample according to the expression levels of immune cell-specific marker genes with R package “gsva.” Most immune cell types related marker genes were obtained from the article published by Bindea et al. (27).

### Cox Regression Analysis

We assessed the impact of immune cell types on clinical survival data and survival time by Cox proportional hazards regression analysis based on the R package “survival” and “forestplot.” Cell types with a high hazard ratio were considered to be risk factors to OS.

### Hub Genes Correlated With m^6^A RNA Methylation Clusters and Immune Infiltration Based on Weighted Correlation Network Analysis (WGCNA)

We extracted all the DEGs (according to adj. *p*-value < 0.01, |logFC| ≥ 2, total = 729) from limma analysis with expression data retrieved from TCGA GBM/LGG datasets to perform Weighted correlation network analysis (WGCNA) using R package “limma.” We applied R package “WGCNA” to find clinical traits-related modules and hub genes among them (28). The adjacency matrix was then transformed into topological overlap matrix (TOM). Genes were divided into different gene modules according to the TOM-based dissimilarity measure. We set soft-thresholding power as 9 (scale free R2 = 0.85), cut height as 0.2, and minimal module size as 30 to identify key modules. Those with gene significance (GS) > 0.5 and module membership (MM) > 0.9 were defined as hub genes.

### Validation of Prognostic Values of Hub Genes

To predict the clinical outcomes of glioma patients with the hub genes, we applied LASSO Cox regression algorithm to the 5 hub genes in the TCGA datasets. We selected four genes to build the risk signature based on the minimum criteria, and the coefficients obtained from the LASSO algorithm were used to calculate the risk score for each patient as follows:

$$\begin{array}{l} Riskscore=\overset{n}{\underset{i=1}{\sum }}*\beta_{i} \end{array}$$

where n was the number of prognostic genes, exp_*i*_ the expression value of gene *i*, and β_*i*_ the regression coefficient of gene *i* in the LASSO algorithm. Using the median risk score as a cutoff value, glioma patients were divided into high- and low-risk score groups. Moreover, the relation between the prognosis signature and OS was investigated based on the external cohort CGGA datasets.

The Kaplan-Meier method was used to assess the differences of overall survival (OS) between low- and high-risk score glioma patients with R package “survival”.

The time-dependent receiver operating characteristic (ROC) curve was used to measure the prognostic performance by comparing the areas under the ROC curves (AUC) using R package “pROC.” 10-fold cross method was applied for ROC validation and AUC value calculation.

All the scripts were uploaded at Github website (https://github.com/mvpsc30/FIO-m6A-immune).

### Assessment of Immunohistochemistry Data

The *PDPN* and *TIMP1* immunohistochemistry results were acquired from the Human Protein Atlas (HPA, https://www.proteinatlas.org/) database (29). The *EMP3* and *TAGLN2* protein levels of selected genes were evaluated through commercially glioma tissue-microarrays and H-scores between Low-grade gliomas and High-grade gliomas.

### Real-Time RT-PCR

Total RNA was extracted from tissue samples and cells using TRIzol reagent (Invitrogen) after washing with PBS. cDNA was synthesized from purified RNA using a SuperScript III First-Strand cDNA synthesis system (18080051, Life Technologies) according to the manufacturer's instruction. SYBR Green PCR Master Mix (Applied Biosystems, CA, USA) was used for PCR amplification and a real-time PCR machine (iQ5, Bio-Rad Laboratories) was used to quantify the expression of mRNAs. β-actin was used as endogenous control and the expression levels were quantified using the methods of 2–ΔΔCt.

Primers:

**Table:** 

	Forward Reverse	
CD68	GGAAATGCCACGGTTCATCCA	TGGGGTTCAGTA CAGAGATGC
YTHDC1	AACTGGTTTCTAAGCCACTGAGC	GGAGGCACTACTTGATAGACGA
WTAP	CATTTTGTGGCAGCGAGACC	AATCCTCTCCAGGCAGAAGC
TIMP1	CTTCTGCAATTCGACCTCGT	ACGCTGGTATAAGGTGGTCTG
PDPN	GTGTAACAGGCATTCGCATCG	TGTGGCGCTTGGACTTTGT

### Cell Culture and Transfection

Human glioma cell line U87 and A172 were acquired from the American Type Culture Collection (ATCC) and cultured in DMEM medium (Gibco, Life Technologies, Grand Island, NY) supplemented with 10% fetal bovine serum (Gibco) and 100 U/ml penicillin/streptomycin (Gibco). According to the manufacturer's instructions, the Lipo 2000 transfection reagent was applied for the transfection. The siRNAs against *TIMP1* (siRNA ID: s14143, ThermoFiher), *PDPN* (EHU119431, Sigma) and negative control (SIC001, Sigma) were purchased.

### Western Blotting

Western blot (WB) assays was performed as previously described (30). Briefly, we prepared cell extracts for Western blotting in RIPA buffer. Then, lysates were separated by SDS-PAGE and were transferred to PVDF membranes (Millipore, Billerica, MA). Primary antibodies *PDPN* (Abcam, ab236529,1:1000), *TIMP1* (Abcam, ab109125,1:1000), *EMP3* (Santa cruz, sc-81797, 1:100), *TAGLN2* (Proteintech, 10234-2-AP, 1:200), and *GAPDH* (Abcam, ab181602, 1:10000) were used along with HRP-labeled secondary antibody (1:10000, Sigma) in Western blot. The immune complex was detected by chemiluminescence (GE Healthcare, Wauwatosa, WI).

### Cell Viability and Cell Death Measurement

Cell viability was measured using the CellTiter-Glo®luminescent cell viability assay (Promega) based on the manufacturer's instructions. For phosphatidylserine exposure, cells were stained with annexin V-PE as instructed by the manufacturer (BD Biosciences, San Jose, CA), and assayed by flow cytometry (CyAn ADP, Beckman Coulter, Brea, CA, USA).

### Statistical Analysis

Experimental results were analyzed with a Student's *t*-test and graphed using Graphpad Prism application (GraphPad Software, Inc., La Jolla, CA). Data are expressed as mean ± *SD*. A *p* < 0.05 was considered with statistical significance. The correlation between the expression profiles of TIMP1 and PDPN with immune and macrophage marker was analyzed using Spearman's rank test.

## Results

### Consensus Clustering of m^6^A RNA Methylation Regulators Identified Two Clusters of Gliomas With Distinct Immune Infiltration

**Figure 1:**
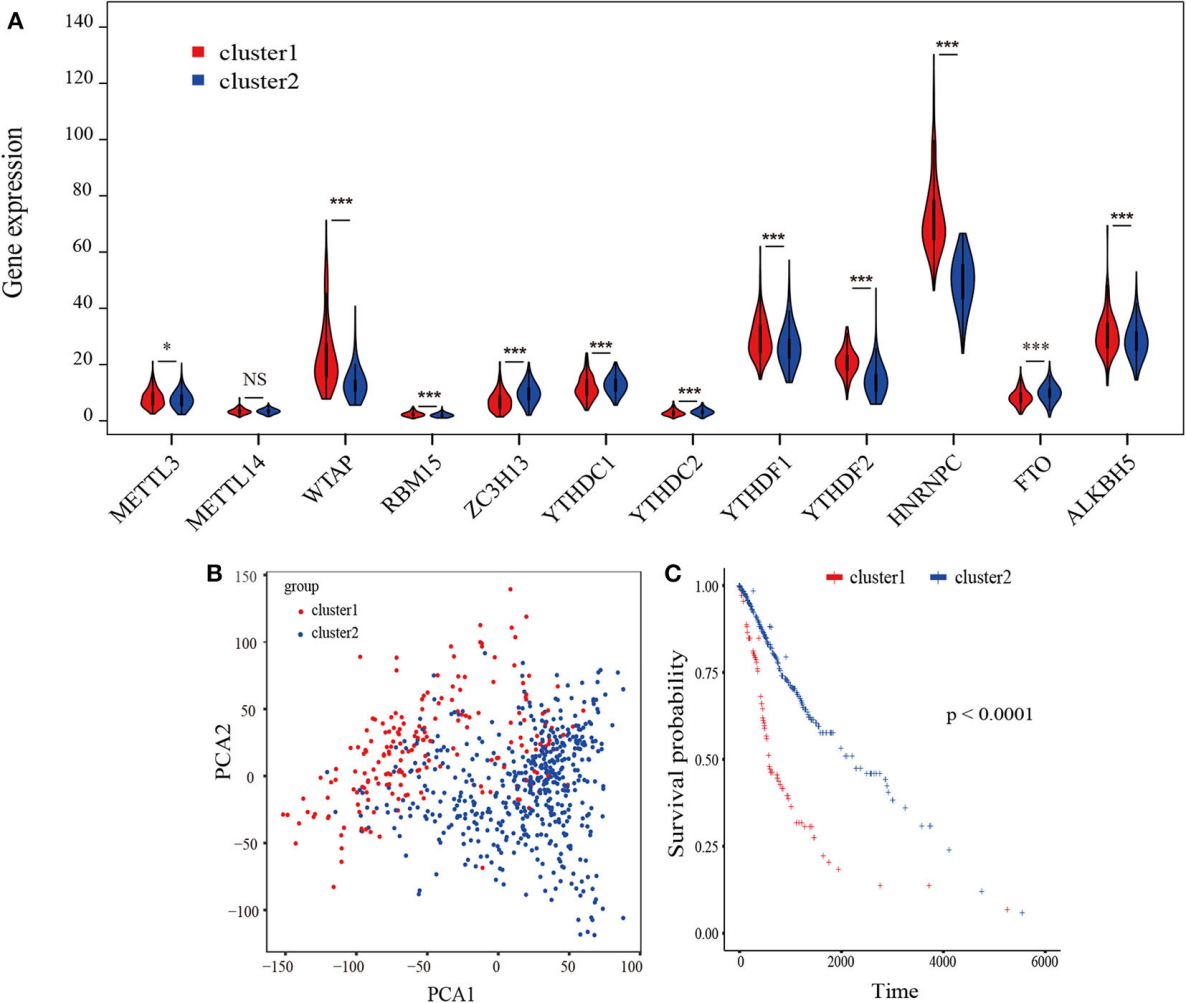
Identification of consensus clusters by m^6^A RNA methylation regulators overall survival of gliomas in the cluster 1/2 subgroups. **(A)** Violin plot of the two clusters (cluster1/2) defined by the m6A RNA methylation regulators consensus expression. **(B)** Principal component analysis of the total RNA expression profiles in the TCGA-GBM/LGG datasets. Gliomas in the cluster1 subgroup are marked with red. **(C)** Kaplan–Meier overall survival (OS) curves for 665 TCGA glioma patients of different cluster. *P < 0.05; **P < 0.01; ***P < 0.001; ****P < 0. 0001.

A flowchart of this study is shown in Supplementary Figure 1. Based on biological functions of each m^6^A RNA methylation regulator in clinical prognosis, we performed consensus clustering based on gene expression of 12 key m^6^A RNA regulators in TCGA datasets. Due to the expression analogy of m^6^A regulators, the clustering analysis would classify the samples into different clusters. After evaluating the relative change in the area under the cumulative distribution function (CDF) curve and consensus heatmap, we selected a three-cluster solution (*K* = 2), which has no obvious increase in the area under the CDF curve (Supplementary Figures 2A–D). To further determine the optimal *K*, two methods (Average Silhouette method and Gap Statistic method) were applied. Based on these methods, two subgroups clustered by *k* = 2, namely, cluster 1 and cluster 2 subgroups were found (Supplementary Figures 2E,F). Most parts of m6A RNA methylation regulators' expressions showed clear distinction and significant difference in two cluster subgroups (Figures 1A,B). In order to better understand the interaction among the 12 m^6^A regulators, we assessed the interaction and correlation among these regulators (Supplementary Figure 3).

The Kaplan-Meier survival analysis revealed a significant shorter OS in cluster 1 subgroup than the cluster 2 subgroup (Figure 1C). Moreover, we analyzed the DEGs between cluster1 and cluster2, and annotated their function Gene Set Variation Analysis (GSVA) for biological processes. The results indicated that DEGs are enriched in immune-related biological processes, including IL2/STAT5, IL6/JAK/STAT3, and Interferon-γ response signaling (Table 2) and the two categories identified by consensus clustering are correlated with immune infiltration of glioma.

**Table 2:** Differences in pathway activities scored per sample by GSVA between cluster 1 and cluster 2, cluster 2 vs. cluster 1.

	**logFC**	**adj.P.Val**
HALLMARK_MYC_TARGETS_V1	−0.53	4.08E-69
HALLMARK_DNA_REPAIR	−0.35	8.65E-59
HALLMARK_E2F_TARGETS	−0.48	1.84E-49
HALLMARK_UNFOLDED_PROTEIN_RESPONSE	−0.29	1.01E-37
HALLMARK_MTORC1_SIGNALING	−0.28	1.84E-33
HALLMARK_GLYCOLYSIS	−0.19	4.81E-27
^*^ HALLMARK_TNFA_SIGNALING_VIA_NFKB	−0.28	2.16E-24
HALLMARK_G2M_CHECKPOINT	−0.29	8.59E-23
HALLMARK_MYC_TARGETS_V2	−0.30	1.45E-21
HALLMARK_P53_PATHWAY	−0.15	1.18E-20
HALLMARK_ALLOGRAFT_REJECTION	−0.25	1.18E-16
HALLMARK_EPITHELIAL_MESENCHYMAL_TRANSITION	−0.18	1.17E-13
HALLMARK_OXIDATIVE_PHOSPHORYLATION	−0.21	3.01E-13
^*^ HALLMARK_INTERFERON_ALPHA_RESPONSE	−0.29	2.45E-12
^*^ HALLMARK_IL6_JAK_STAT3_SIGNALING	−0.20	1.26E-11
^*^ HALLMARK_TGF_BETA_SIGNALING	−0.16	9.81E-11
HALLMARK_ANDROGEN_RESPONSE	−0.12	7.44E-10
HALLMARK_PEROXISOME	−0.11	2.67E-09
^*^ HALLMARK_INTERFERON_GAMMA_RESPONSE	−0.19	3.11E-09
HALLMARK_IL2_STAT5_SIGNALING	−0.13	2.72E-08

* *Pathways related to immune response are marked with asterisk*.

### Immune Landscape Was Significantly Associated With m^6^A RNA Methylation Regulators

**Figure 2:**
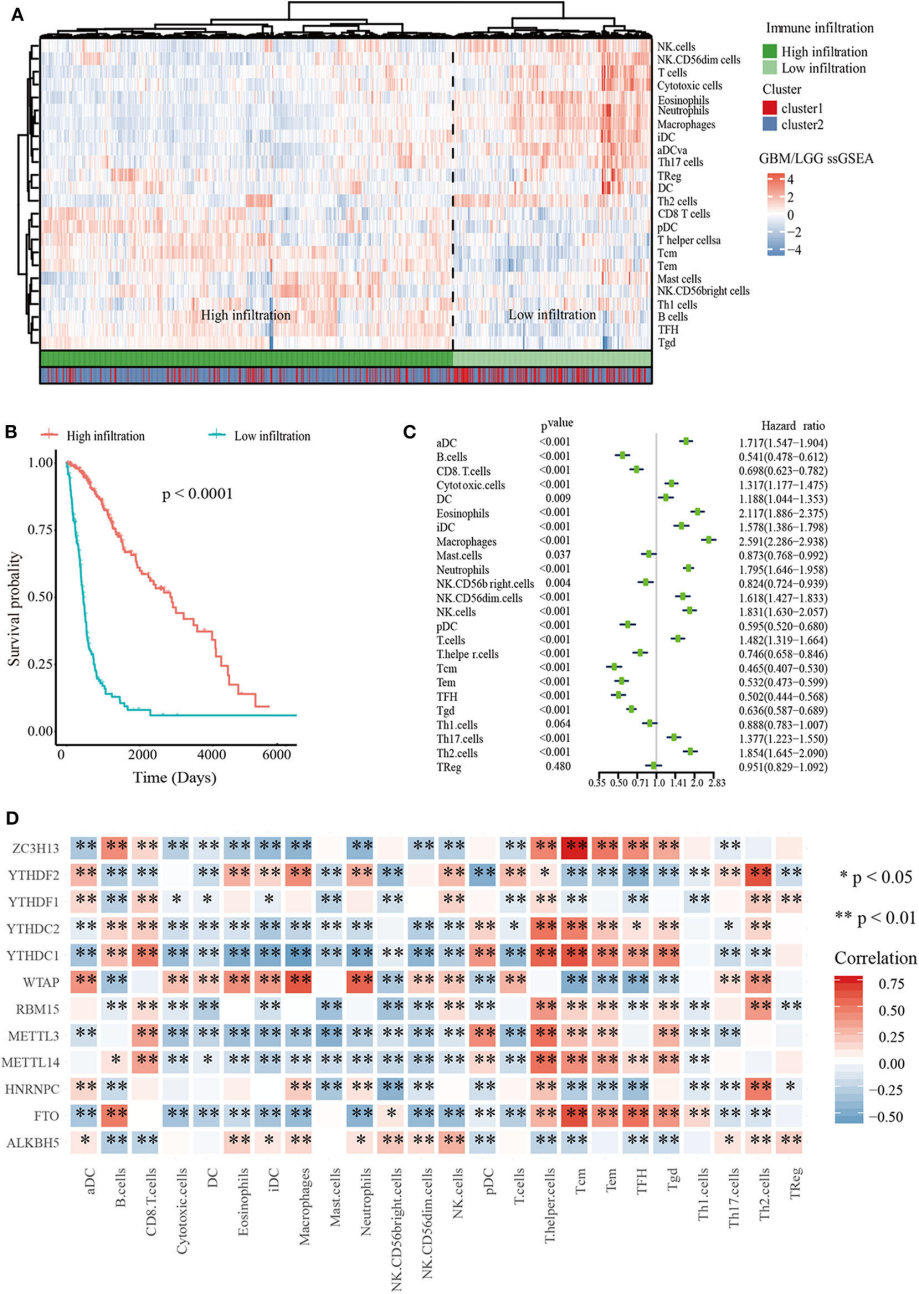
Immune landscape of glioma. **(A)** Heatmap of ssGSEA scores of TCGA-GBM/LGG and Table of cluster and immune infiltration subgroups (Chi-square test: X-squared = 116.63, *p* < 2.2e−16). **(B)** Kaplan–Meier overall survival (OS) curves for 665 TCGA glioma patients of different immune infiltration subgroups. **(C)** Forest plot for immune cells. The hazard ratios (HR), 95% confidence intervals (CI) calculated by univariate Cox regression are shown. **(D)** Mine plot of relationships between 12 m^6^A methylation regulators and 24 immune cells (*P < 0.05, **P < 0.01).

To explore the roles of immune cells in the malignant progression of gliomas, the RNA-seq data of 665 patients with gliomas from TCGA-GBM/LGG datasets were analyzed to evaluate the immune landscape. The high and low immune infiltration were defined by Euclidean distance and the ssGSEA scores of immune cells. The results indicated that B cells, Tcm cells, and T helper cells were enriched in high immune infiltration glioma. Relatively, gliomas with low infiltration were characterized for macrophages, eosinophils, neutrophils, and aDC cells (Figure 2A).

In order to analyze the relationship between m^6^A cluster group and immune infiltration, Chi-squared test was carried out (*p* < 2.2 × 10−16, Figure 2A). Moreover, we compared the immune infiltration score between cluster 1 and cluster 2, indicating that the proportion of most immune cells types was significantly different between clusters 1 and 2 (Supplementary Figure 4). Then Kaplan-Meier survival curve analysis was performed to explore the roles of immune cell infiltration on the prognosis of patients with glioma. The results revealed that patients with low immune infiltration had worse OS compared with patients with high immune infiltration (Figure 2B). We also applied a univariate Cox regression analysis on the immune cells of TCGA datasets, and found that 23/24 cell types were significantly correlated with OS (*P* < 0.05). Among these 23 immune cells, aDC, DC, iDC, cytotoxic cells, Eosinophils, Macrophages, Neutrophils, NK.CD56dim cells, NK cells, T cells, Th17 cells, and Th2 cells are risky immune cells with HR > 1, while CD8 T cells, B cells, Mast cells, NK.CD56bright cells, pDC, Tem, Tcm, T helper cells, TFH, Tgd, and Th1 cells were protective immune cells with HR < 1 (Figure 2C).

To further determine the relationship between m^6^A RNA methylation regulators and immune cell infiltration, we assessed the relationships between the expressions of m^6^A RNA methylation regulators and immune cells infiltration subgroups. The results indicated that high immune infiltration was strongly related to higher expressions of FTO, MELLT14, METTL3, YTHDC1, YTHDC2, and ZC3H13. Correspondingly, low immune infiltration with higher expressions of ALKBH5, HNRNPC, WTAP, YTHDF1, and YTHDF2 (Supplementary Figure 5). Then we calculated the relationships between each m^6^A RNA methylation regulators and immune cells, revealing that FTO, ZC3H13, and YTHDC1 had a significant positive correlation with Tcm cells. Meanwhile, macrophages had a negative relationship with FTO and ZC3H13 (Figure 2D). These data indicated that m^6^A clusters were highly associated with immune infiltration.

### WGCNA and Identification of the Key Module

In order to explore the key genes that were mostly associated with m6A and immune cell infiltration subtypes in glioma, we performed WGCNA on the TCGA-GBM/LGG datasets. Glioma sample information such as age, m6A cluster subgroups, immune infiltration subgroups, OS and OS status were retrieved from TCGA-GBM/LGG (Supplementary Figure 6A). Eventually identified 6 modules by setting soft-thresholding power as 9 (scale-free R2 = 0.85) and cut height as 0.2 (Supplementary Figures 6B,C). From the heatmap of module-trait correlations, we evaluated that the black module was the most highly related to clinical traits (Supplementary Figure 6D), especially the immune infiltration and outcomes (correlation coefficient = −0.86 and 0.5, *P* = 4E-206 and 1E-39; respectively, Supplementary Figures 6E–G). Lastly, we selected 5 hub genes (TAGLN2, PDPN, TIMP1, EMP3, CHI3L1) from the black module by setting module membership (MM) >0.9 and gene significance (GS) >0.5. These genes were closely related to each other Supplementary Figure 6H).

### Association of Hub Genes With m^6^A RNA Methylation Regulators and Immune Infiltration

We explored the relationship between the expression levels of five hub genes and m^6^A RNA methylation regulators to elucidate the underlying mechanisms of abnormal up-regulation in glioma. The correlation analysis showed that the expression of many hub genes was significantly correlated with m^6^A RNA methylation regulators (Supplementary Figure 7). Additionally, we found that TAGLN2, PDPN, EMP3, and CHI3L1 were positively associated with WTAP (Supplementary Figure 7), while TIMP1 was negatively correlated with YTHDC1.

Then we utilized the Spearman method to study the potential relationship between the expression of glioma hub genes and infiltration of immune cells. Interestingly, hub genes were all positively associated with Macrophages (Supplementary Figure 8). Conversely, negative relationship was observed between these five genes and the infiltration of B cells, Tcm cells and Tem cells (Supplementary Figure 8). These data indicated that the selected five hub genes were highly correlated with m^6^A RNA regulators and immune infiltration.

### Validation of Hub Genes in Datasets

**Figure 3:**
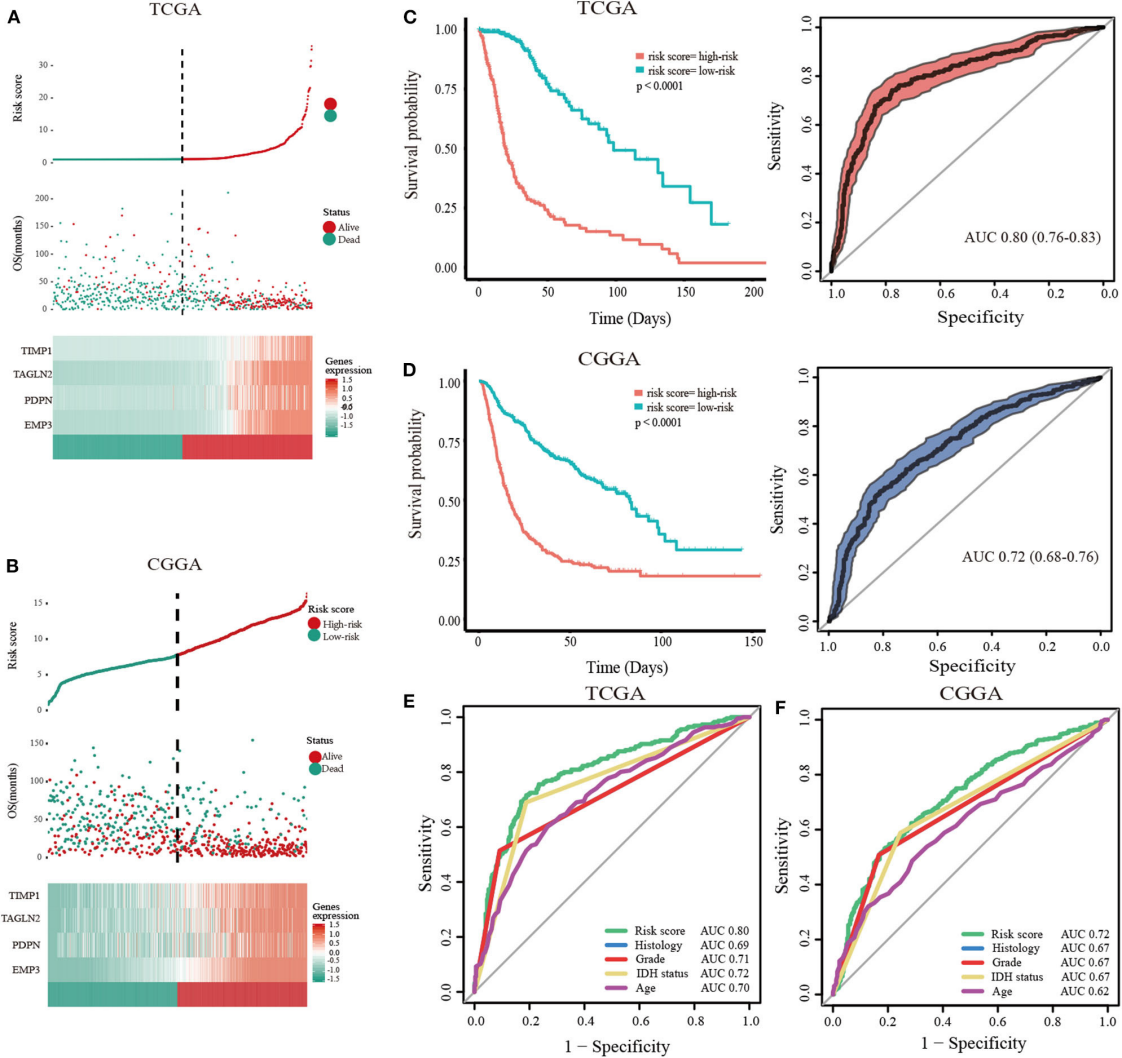
Validation of hub genes highly correlated with clinical traits. In each dataset, the risk score distribution, gene expression profiles, and patients' survival status are displayed (**A**, TCGA; **B**, CGGA). The black-dotted line represents the median cut-off, dividing patients into high- and low-risk groups. Kaplan-Meier and ROC curves with 95% confidence interval for the 4-gene signature in the four datasets. Patients with high risk scores had poor outcome in terms of overall survival (**C**, TCGA; **D**, CGGA). ROC curves comparing prognostic accuracy of risk score with clinical histology, grade, IDH status, and age in internal validation, and external validation cohorts (**E**, TCGA; **F**, CGGA).

To predict the clinical outcomes of glioma with the hub genes, we applied the LASSO Cox regression algorithm to the five hub genes in the TCGA datasets (Supplementary Figures 9A,B). Four genes were highly associated with clinical features, such as grades, transcriptome subtype and IDH status (Supplementary Figures 10A–C). Moreover, these four genes were used to set up the risk signature based on the minimum criteria. Next, to assess the differences of survival time between low- and high-risk glioma patients, the Kaplan-Meier method was performed. Meanwhile, the log-rank test was also used to determine the statistical significance between groups. The time-dependent ROC curve was employed to measure the prognostic performance by comparing the AUC. Compared with those in the low-risk group, we illustrated that the glioma patients in the high-risk group had shorter OS, (Figures 3A,B, TCGA: HR = 1.07, 95% CI = 1.06–1.08, *P* < 0.01; CGGA: HR = 1.19, 95% CI = 1.16–2.23, *P* < 0.01). The time-dependent ROC curves revealed that the AUC for the 4-gene signature achieved 0.80 (0.76–0.83) and 0.72 (0.68–0.76) for the OS in TCGA and CGGA datasets, respectively (Figures 3C,D). Furthermore, the risk score exhibited a higher prognostic accuracy for OS than clinical histology, grade, IDH status and age (Figures 3E,F). These findings suggested an effective performance for predicting OS for glioma patients.

### Validation the Expression and Function of TIMP1 and PDPN

**Figure 4:**
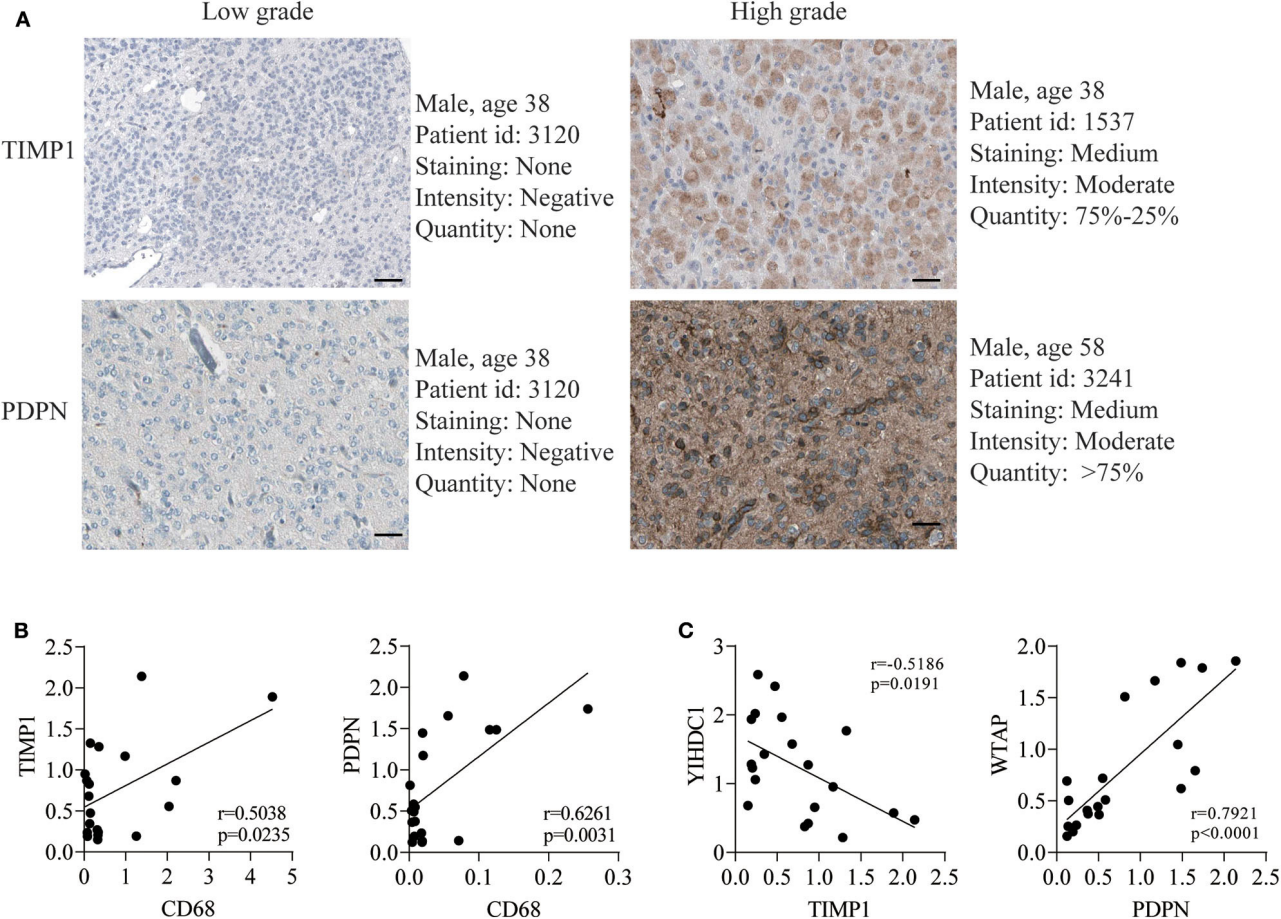
TIMP1 and PDPN reveal its higher expression in GBM and malignant biological phenotype *in vitro*. **(A)** The translation expression level of the TIMP1 and PDPN were positively correlated with disease status as they were upregulated in gliomas samples. **(B)** The relationship of TIMP1 and PDPN with macrophage marker CD68. **(C)** The relationship of TIMP1 and PDPN with indicated m6A gene.

To further validate the expression of four genes in gliomas, we next detected their expressions in The Human Protein Atlas database, and the results revealed the PDPN and TIMP1 were higher expression in high-grade gliomas (Figure 4A). In addition, TAGLN2 and EMP3 were performed in commercially glioma tissue-microarrays. The H-score of both proteins was not statistically significant between low and high-grade gliomas (Supplementary Figure 11). Moreover, in the correlation analysis, we uncovered that TIMP1 and PDPN were positively correlated with marker genes of macrophage (Figure 4B, Table 3 and Supplementary Figure 8). TIMP1 was negatively related with YHDC1, while PDPN was positively related with WTAP (Figure 4C and Supplementary Figure 7). By knockdown the expression of PDPN or TIMP1, the cell proliferation was decreased, and the apoptosis and necrosis were increased in U87 and A172 (Supplementary Figure 12).

**Table 3:** Clinical data of patients.

**Case**	**Sex**	**Age**	**Tumor volume (cm^**3**^)**	**Grade**	**Application**
1	F	56	12.1	1	PCR
2	M	46	8.42	1	PCR
3	F	44	21.1	1	PCR
4	F	64	25.2	1	PCR
5	M	41	23.1	1	PCR
6	F	67	31.8	1	PCR
7	F	45	33.4	1	PCR
8	F	66	31.6	1	PCR
9	M	70	26.1	1	PCR
10	M	54	12.9	1	PCR
11	M	70	21.1	1	PCR
12	M	33	35.4	1	PCR
13	F	55	33.1	4	PCR
14	F	76	46.1	4	PCR
15	M	33	32.7	4	PCR
16	F	71	27.8	4	PCR
17	M	62	36.0	4	PCR
18	F	59	33.5	4	PCR
19	M	59	42.1	4	PCR
20	F	60	26.9	4	PCR

## Discussion

As the most aggressive primary brain tumor, glioma is considered as an enigma in neurosurgery (31, 32). Advanced knowledge of its genomic changes has promoted the discovery of prognostic signatures to facilitate the personalized treatment decisions (33–35). However, no previous studies have investigated the efficacy of the combination of m^6^A and immune infiltration. Here, we developed and validated a novel 4-gene prognostic model based on the combination of m^6^A RNA methylation and landscape of immune microenvironment. The developed 4-gene signature was able to identify the glioma patients with different risk levels for prognosis, which may compensate the already known prognostic indicators, such as age, tumor grade or histology. Additionally, we confirmed that *PDPN* and *TIMP1* were higher expressed in high-grade glioma, and the Pearson correlation validated that *PDPN* and *TIMP1* were correlated with marker gene of macrophage and indicated m6A gene.

m^6^A, the most prevalent intra-mRNA modification, is required for post-transcriptional regulation of mRNA in various cell types (11, 12, 36). Previous studies have shown that m6A could be a signature for predicting the prognosis in different type of cancers, such as renal cell carcinoma, hepatocellular carcinoma, bladder cancer and head and neck squamous cell carcinoma (37–40). We found that WTAP and HNRNPC were significantly increased in cluster 1 than cluster 2 (Figure 2). In the GBM, WTAP was found to be overexpressed and regulate migration and invasion *in vitro* (41). Its high expression was associated with poor postoperative survival (42). In addition, HNRNPC could also control the aggressiveness of GBM cells and be regarded as the potential prognostic biomarker and therapeutic targets of GBM (43).

With the high-speed development of omics, high-throughput tumor databases have been established, including TCGA and CGGA, which provided a solid foundation for analyzing the RNA modification and microenvironments of glioma (3, 44–46). One of the emerging strategies of management is based on the roles of immune cells in the growth and maintenance of tumors (47). According to the recent studies, myeloid-derived suppressor cells (MDSC) and tumor-associated macrophages (TAMs) have been identified as promising targets for anti-cancer treatment (48, 49). Neoantigen-targeting vaccines have also increased tumor-infiltrating T cells and altered the immune milieu of glioblastoma (50). According to the TCGA database, Jia et al. has drawn a list of 44 tumor microenvironment related genes and proved them in an independent GBM cohort as potential biomarkers for GBM (51). However, the outcomes may lead to the discordance generally based on only one factor (51, 52). In our current study, we integrated m6A and immune infiltration in TCGA to build a model to improve the overall prediction of outcome for patients with glioma. Four survival-related genes (*TAGLN2, PDPN, TIMP1*, and *EMP3*) were identified and verified by four external datasets. These combination of these four genes provided a more reliable signature, relative to that extracted from a single dataset. Furthermore, *PDPN* and *TIMP1* were confirmed that they were higher expression in high-grade glioma and knockdown their expression decreased the glioma cell proliferation *in vitro*.

*TAGLN2* is considered as a smooth muscle cytoskeletal protein (53). It has been proposed to be associated with growth and migration in bladder cancer (54, 55), esophageal squamous cell carcinoma (56), and gliomas (57). Moreover, it's up-regulation is associated with tumorigenesis and tumor progression (54, 58). Silence of *TAGLN2* in gliomas cell lines significantly inhibited invasion and tumor growth (57). Increased expression of *TAGLN2* was correlated with deteriorative tumor grade, and the function and regulation made it as a candidate prognostic biomarker (57). Jin et al. has also shown *TAGLN2* as a potential biomarker of tumor-derived lung-cancer endothelial cells (59). Another study demonstrated that *TAGLN2* could be a prospective tumor tissue marker for diagnosis and evaluating lymph node metastasis in bladder cancer patients (60).

*EMP3* belongs to the PMP-22/EMP/MP20 family, which is thought to be involved in cell proliferation, cell-cell interactions and function as a tumor suppressor. Alaminos et al. have suggested that *EMP3* was associated with poor survival (61). *EMP3* overexpression in breast cancer was related to stronger HER-2 expression that may indicate a novel therapeutic target (62). Ma et al. have demonstrated that *EMP3*-mediated miR-663a inhibits the gallbladder cancer progression via the MAPK/ERK pathway (63). Recently, the bioinformatics analysis also found that *EMP3* was one of the validated gene panel independently and was correlated with the GBM survival (64, 65). Another bioinformatics analysis though significant analysis of microarray (SAM) identified that *EMP3* could be used to estimate glioma patient prognosis (66).

*TIMP* metallopeptidase inhibitor 1 (*TIMP1*) is a glycoprotein which antagonized mostly known MMPs. The encoded protein can promote cell proliferation in many cell types and may also have an anti-apoptotic function. A high serum level was found as a poor prognostic indicator in GBMs (67). *TIMP1* has been suggested to interact with *P75NTR* in metastatic carcinoma and glioma cells (68), and silence of *TIMP1* or inhibition of NF-kappa B activity led to slower tumor growth *in vivo* (69). Several studies have shown that *TIMP1* was an important part of prognosis model and could be a biomarker for diagnosis (70–72). Furthermore, Jackson et al. have reviewed that *TIMP1* overexpression is consistently correlated with cancer progression or poor prognosis (73).

Podoplanin (*PDPN*) is a transmembrane receptor that participates in various physiological and pathological processes, such as cell motility, tumor metastasis and angiogenesis (74–76). It regulated mammary stem cell function that reduced mammary tumor formation in breast cancer and could be a new regulator of Wnt/β-catenin signaling (77). PDPN receptor are upregulated in cancer cells, immune cells, synoviocytes, and fibroblasts that increase tissue inflammation and invasion to promote both arthritis and cancer (78). *PDPN*-expressing macrophages (PoEMs) stimulated local matrix remodeling, and macrophage-specific *PDPN* knockout restrained lymphangiogenesis and reduced lymphatic cancer spread (79). *PDPN*-positive cancer-associated fibroblasts (CAFs) contributed to an essential role in primary resistance to epidermal growth factor receptor tyrosine kinase inhibitors (EGFR-TKI) (80). Moreover, *PDPN* has been considered as a novel biomarker, chemotherapeutic target and a target for CAR T-cell therapy that may be a potential adoptive immunotherapy to treat GBM (81, 82).

Our finding provides a novel insight into the relationship between m6A and immune infiltration, and we laid a solid foundation for four genes that could be a new prognosis indicator for gliomas patients. In addition, we also developed a user-friendly R shiny web app (http://www.houshixu.cn:3838/sample-apps/fio/) for easier usage. Remarkably, several limitations should be noted. In this study, prognostic factors were found by combining m6A and immune microenvironment. However, we do not have large quantities of samples to verify them and the clustering of glioma by m6A regulators is probably skewed by the grade of glioma. Whether *TAGLN2* and *EMP3* modulate cell proliferation were unclear. Moreover, the signature requires further validation in prospective studies and multicenter clinical trials.

## Conclusions

We construct a novel prognostic model that provides new insights into glioma prognosis. The *PDPN* and *TIMP1* may serve as potential biomarkers for prognosis of glioma.

## Data Availability Statement

Publicly available datasets were analyzed in this study, these can be found in The Cancer Genome Atlas via the University of California Santa Cruz (UCSC) Xena browser (https://xenabrowser.net/datapages/).

## Ethics Statement

The studies involving human participants were reviewed and approved by Shanghai General Hospital, Shanghai Jiao Tong University School of Medicine, Shanghai 200080, China. The patients/participants provided their written informed consent to participate in this study.

## Author Contributions

This work was carried out in collaboration with all authors. ML, MW, and TM contributed to the conception and design of the study. SL and HX carried out the experiments. SL, HX, and AZ contributed to all figures and tables. SL, TM, MW, and YN revised the manuscript. SL, HX, AZ, and YX contributed to data collection and analysis. All authors have read and approved the final manuscript. All authors contributed to the article and approved the submitted version.

## Conflict of Interest

The authors declare that the research was conducted in the absence of any commercial or financial relationships that could be construed as a potential conflict of interest.

## Supplementary Material

Supplementary Figure 1 An overall flowchart of this work.

Supplementary Figure 2 Identification of consensus clusters by m6A RNA methylation regulators. Consensus clustering matrix for *k* = 2 to *K* = 5 **(A–D)**. **(E)** Consensus clustering cumulative distribution function (CDF) for *k* = 2–9. **(F)** Relative change in area under CDF curve for *k* = 2–5. Silhouette analysis **(G)** and Gap analysis **(H)** showed that 2 clusters were appropriate classification for the data.

Supplementary Figure 3 Spearman correlation analysis of the 12 m^6^A modification regulators.

Supplementary Figure 4 The immune infiltration score between cluster 1 and cluster 2.

Supplementary Figure 5 Relationship between m^6^A RNA methylation regulators and immune infiltration **(A–J)** violin plot for 12 m^6^A regulators and immune infiltration subgroups.

Supplementary Figure 6 Identification of key modules correlated with clinical traits in the TCGA-GBM/LGG datasets through WGCNA. **(A)** Clustering dendrograms of genes. Color intensity varies positively with age, m^6^A cluster subgroups, immune infiltration subgroups, overall survival and overall survival status. Analysis of the scale-free fit index **(B)** and the mean connectivity **(C)** for various soft-thresholding powers. **(D)** Heatmap of the correlation between module eigengenes and clinical traits of diffuse gliomas. Each cell contains the correlation coefficient and *P*-value. **(E)** Dendrogram of all DEGs clustered based on a dissimilarity measure (1- TOM). **(F)** Clustering of module eigengenes. The red line indicates cut height (0.2). **(G)** Scatter plot of module eigengenes in the black module. **(H)** Hub genes show strong associations with each other. Red and blue colors indicate positive and negative coefficients and labels from−1 to 1 indicate correlation strength.

Supplementary Figure 7 Association of hub genes' expression with 12 m6A RNA methylation regulators in gliomas. **(A)** TAGLN2 **(B)** PDPN **(C)** TIMP1 **(D)** EMP3 **(E)** CHI3L1.

Supplementary Figure 8 Association of hub genes' expression with immune infiltration cells in gliomas. **(A)** TAGLN2 **(B)** PDPN **(C)** TIMP1 **(D)** EMP3 **(E)** CHI3L1.

Supplementary Figure 9 **(A,B)** The process of building the risk scores containing 4 hub genes and the coefficients calculated by least absolute shrinkage and selection operator (LASSO) Cox regression algorithm are shown.

Supplementary Figure 10 Expression of 4 hub genes in gliomas with different clinicopathological features, from right to left, TIMP1, TAGLN2, PDPN, and EMP3, respectively. **(A)** The expression levels of 4 hub genes in gliomas with different WHO grades. **(B)** The expression levels of 4 hub genes in gliomas with different transcriptome subtypes. **(C)** The expression levels of 4 hub genes in gliomas with different IDH status.

Supplementary Figure 11 H-score of EMP3 **(A)** and TAGLN2 **(B)** of glioma tissue-microarrays.

Supplementary Figure 12 **(A,D)** Western blot analysis validated the knockdown of TIPM1 or PDPN in U87 and A172 cells. **(B,E)** Cell proliferation was determined by ATP assay. **(C,F)** Flow cytometric analysis of Annexin V/PI staining in U87 and A172 cells after transfection with 50 nmol/L siTIMP1 or siPDPN and siCtrl for 72 h.
