# Supplementary figures and images for: Prognosis Analysis and Validation of m6A Signature and Tumor Immune Microenvironment in Glioma
Source: Front Oncol. 2020 Oct 5;10:541401. doi: 10.3389/fonc.2020.541401 (PMC7571468; doi:10.3389/fonc.2020.541401)

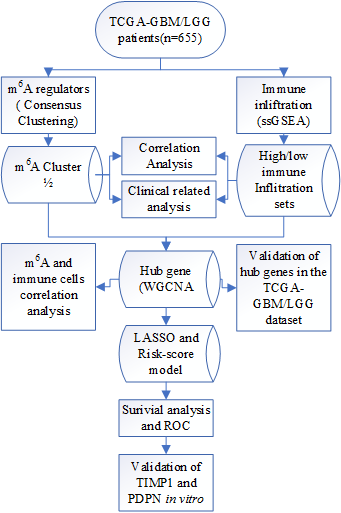

Supplement: Supplementary Figure 1 — An overall flowchart of this work. [file Image_1.TIF]

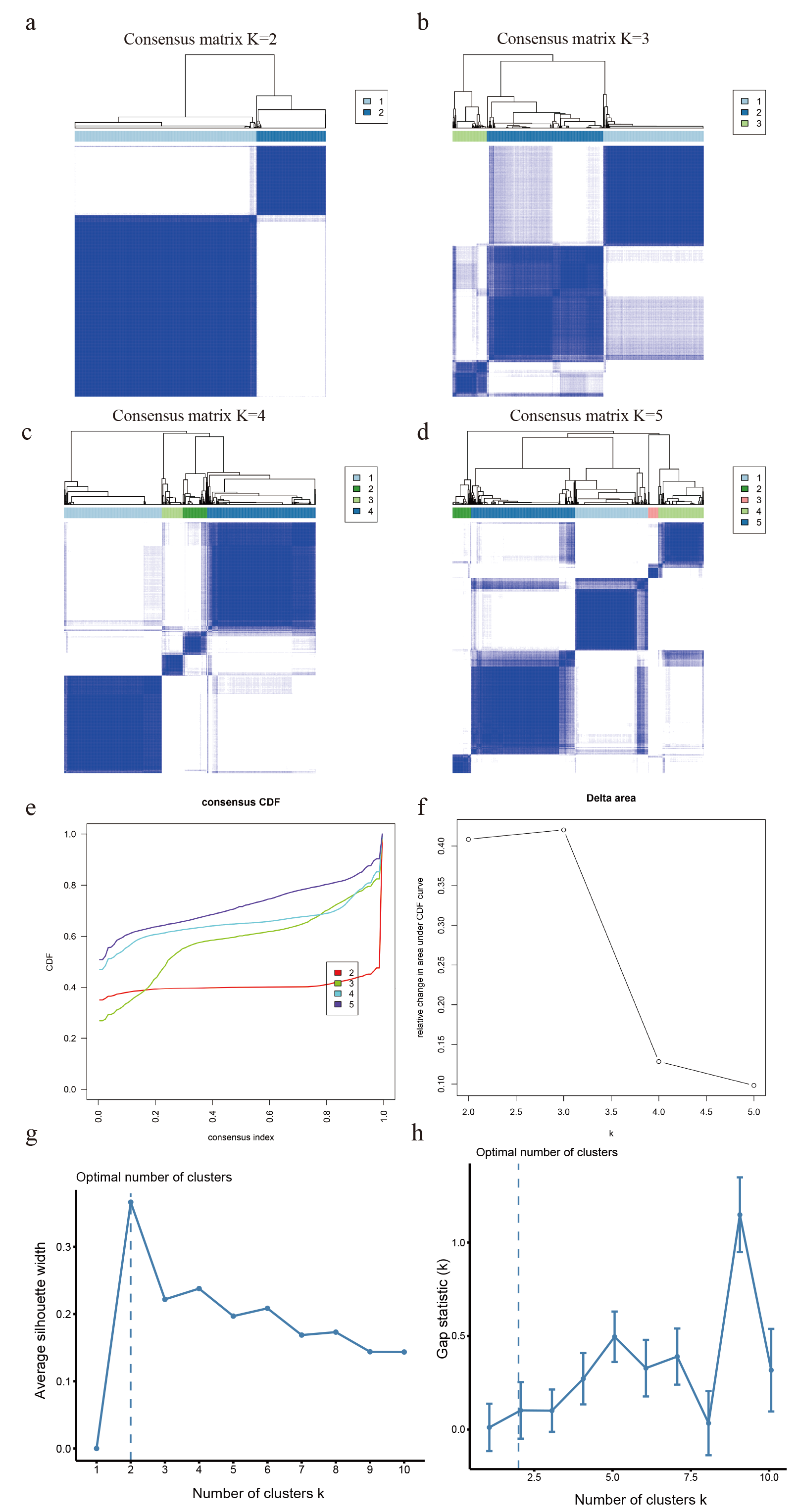

Supplement: Supplementary Figure 2 — Identification of consensus clusters by m6A RNA methylation regulators. Consensus clustering matrix for k = 2 to K = 5 (A–D). (E) Consensus clustering cumulative distribution function (CDF) for k = 2–9. (F) Relative change in area under CDF curve for k = 2–5. Silhouette analysis (G) and Gap analysis (H) showed that 2 clusters were appropriate classification for the data. [file Image_2.TIF]

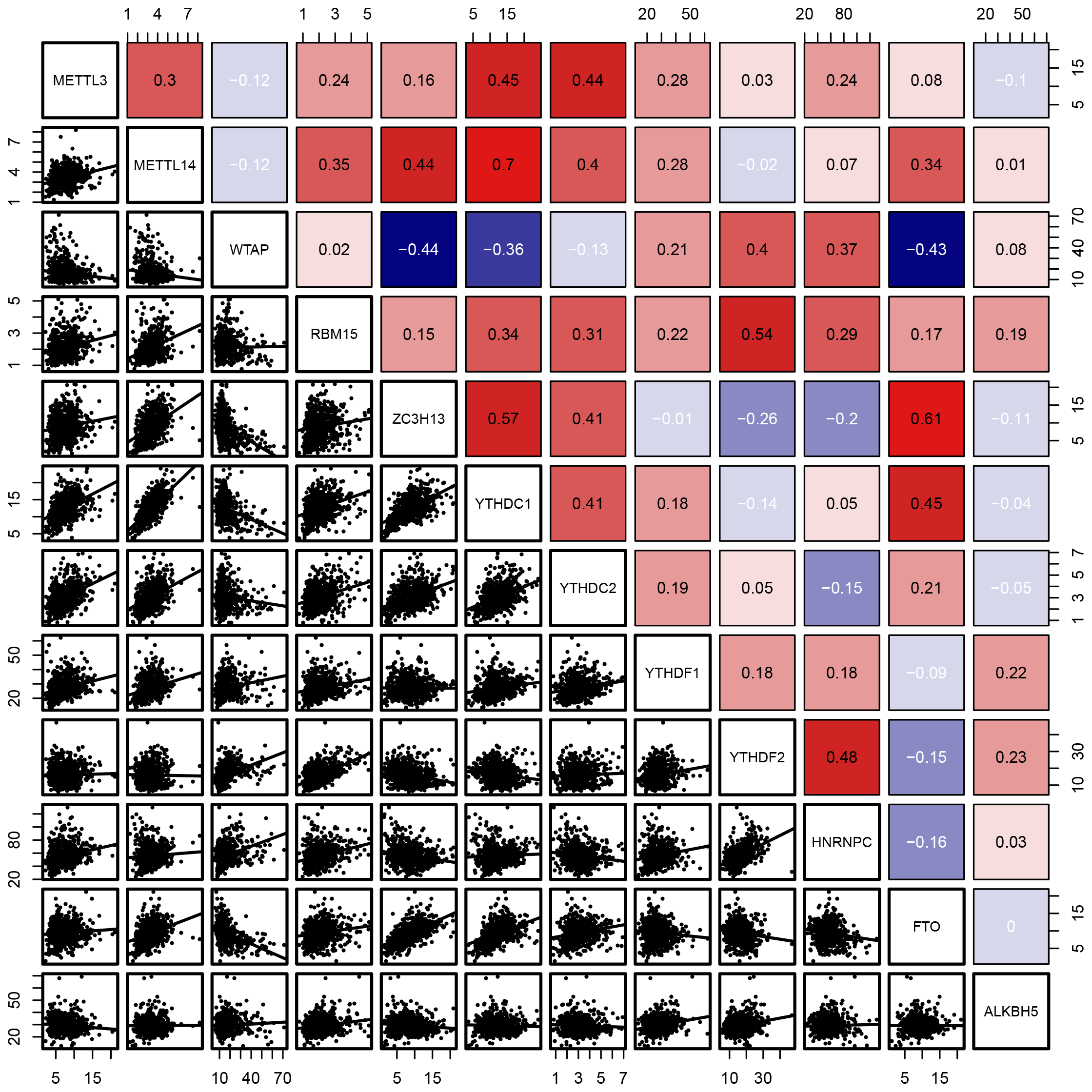

Supplement: Supplementary Figure 3 — Spearman correlation analysis of the 12 m6A modification regulators. [file Image_3.TIF]

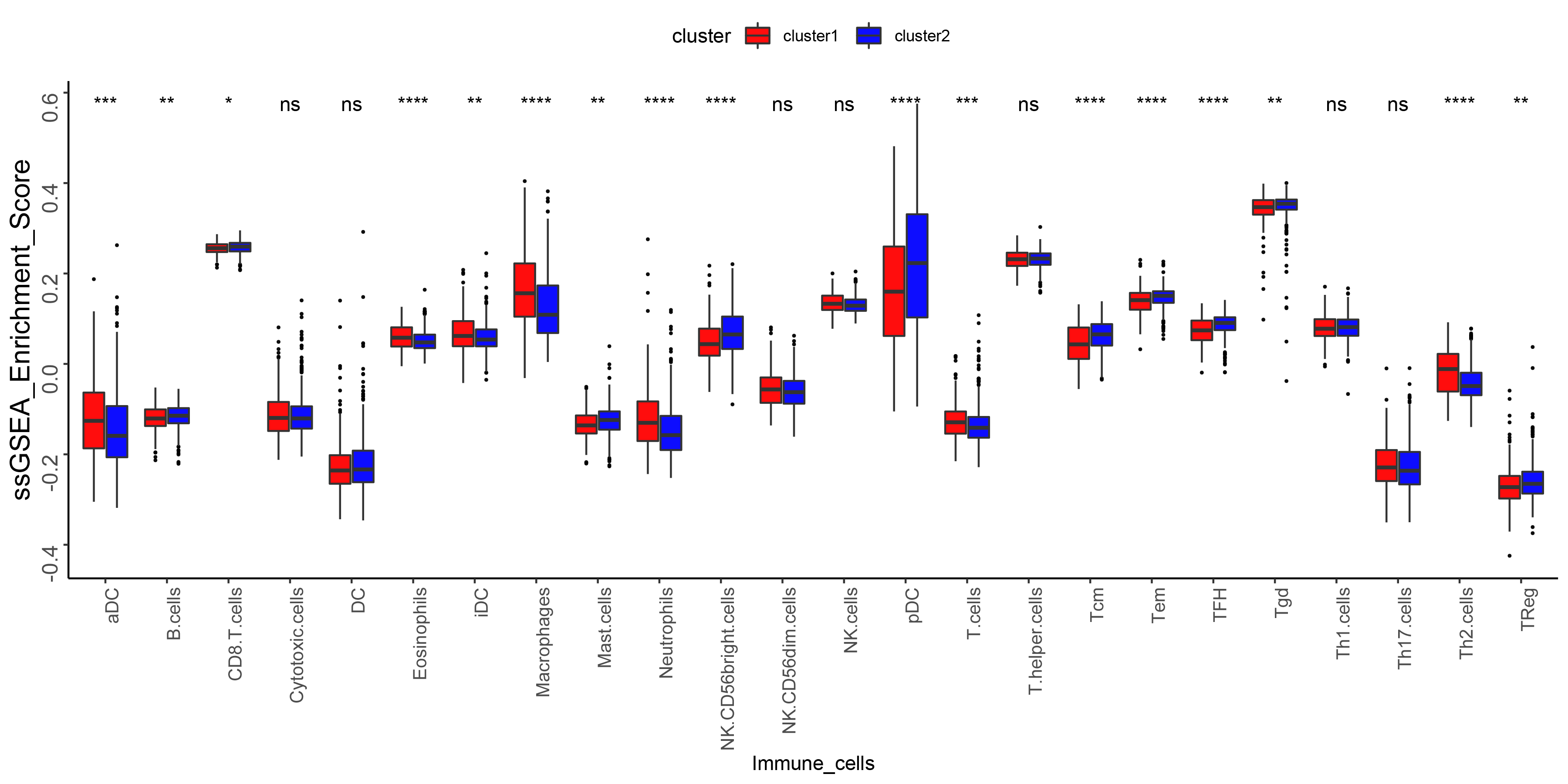

Supplement: Supplementary Figure 4 — The immune infiltration score between cluster 1 and cluster 2. [file Image_4.TIF]

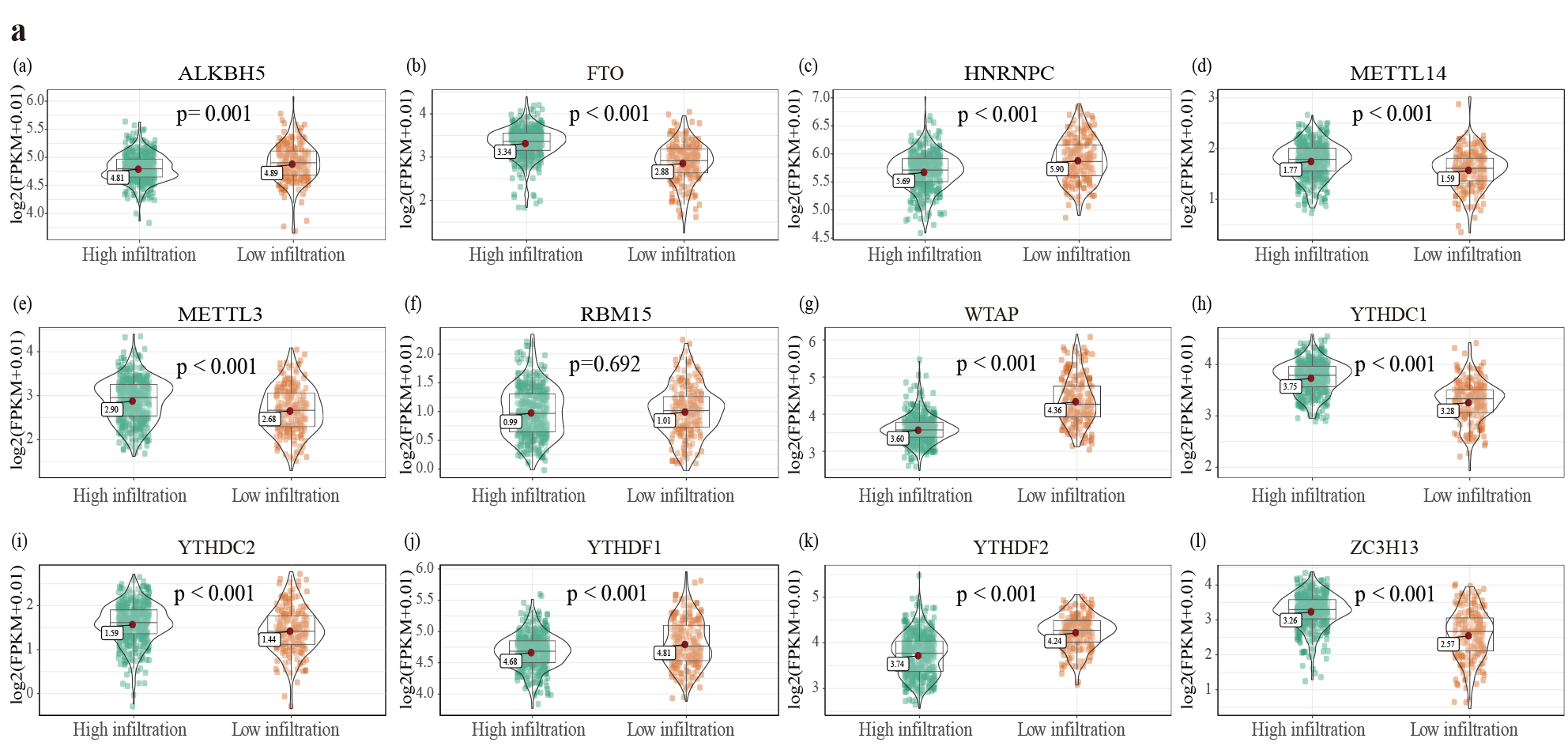

Supplement: Supplementary Figure 5 — Relationship between m6A RNA methylation regulators and immune infiltration (A–J) violin plot for 12 m6A regulators and immune infiltration subgroups. [file Image_5.TIF]

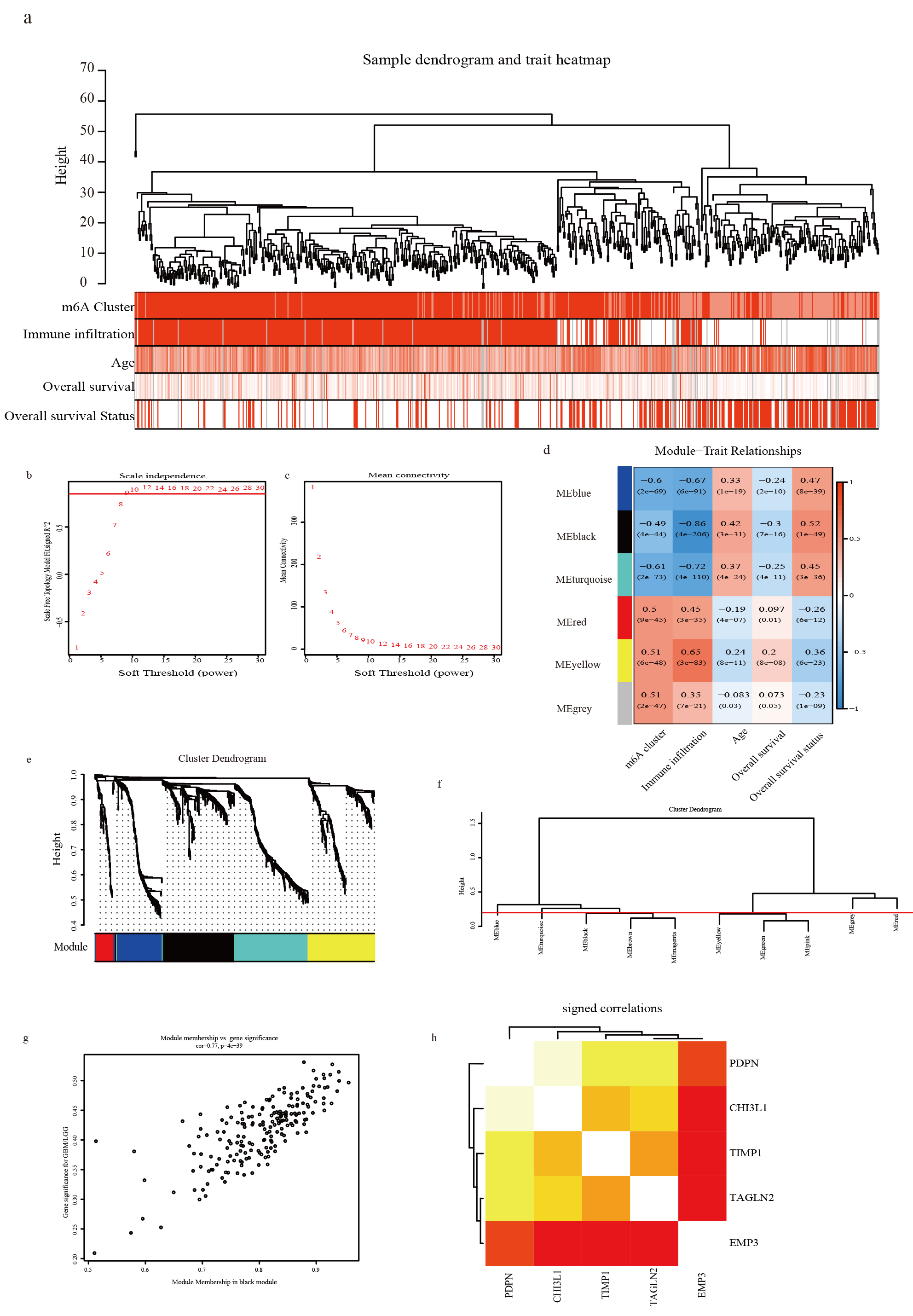

Supplement: Supplementary Figure 6 — Identification of key modules correlated with clinical traits in the TCGA-GBM/LGG datasets through WGCNA. (A) Clustering dendrograms of genes. Color intensity varies positively with age, m6A cluster subgroups, immune infiltration subgroups, overall survival and overall survival status. Analysis of the scale-free fit index (B) and the mean connectivity (C) for various soft-thresholding powers. (D) Heatmap of the correlation between module eigengenes and clinical traits of diffuse gliomas. Each cell contains the correlation coefficient and P-value. (E) Dendrogram of all DEGs clustered based on a dissimilarity measure (1- TOM). (F) Clustering of module eigengenes. The red line indicates cut height (0.2). (G) Scatter plot of module eigengenes in the black module. (H) Hub genes show strong associations with each other. Red and blue colors indicate positive and negative coefficients and labels from−1 to 1 indicate correlation strength. [file Image_6.TIF]

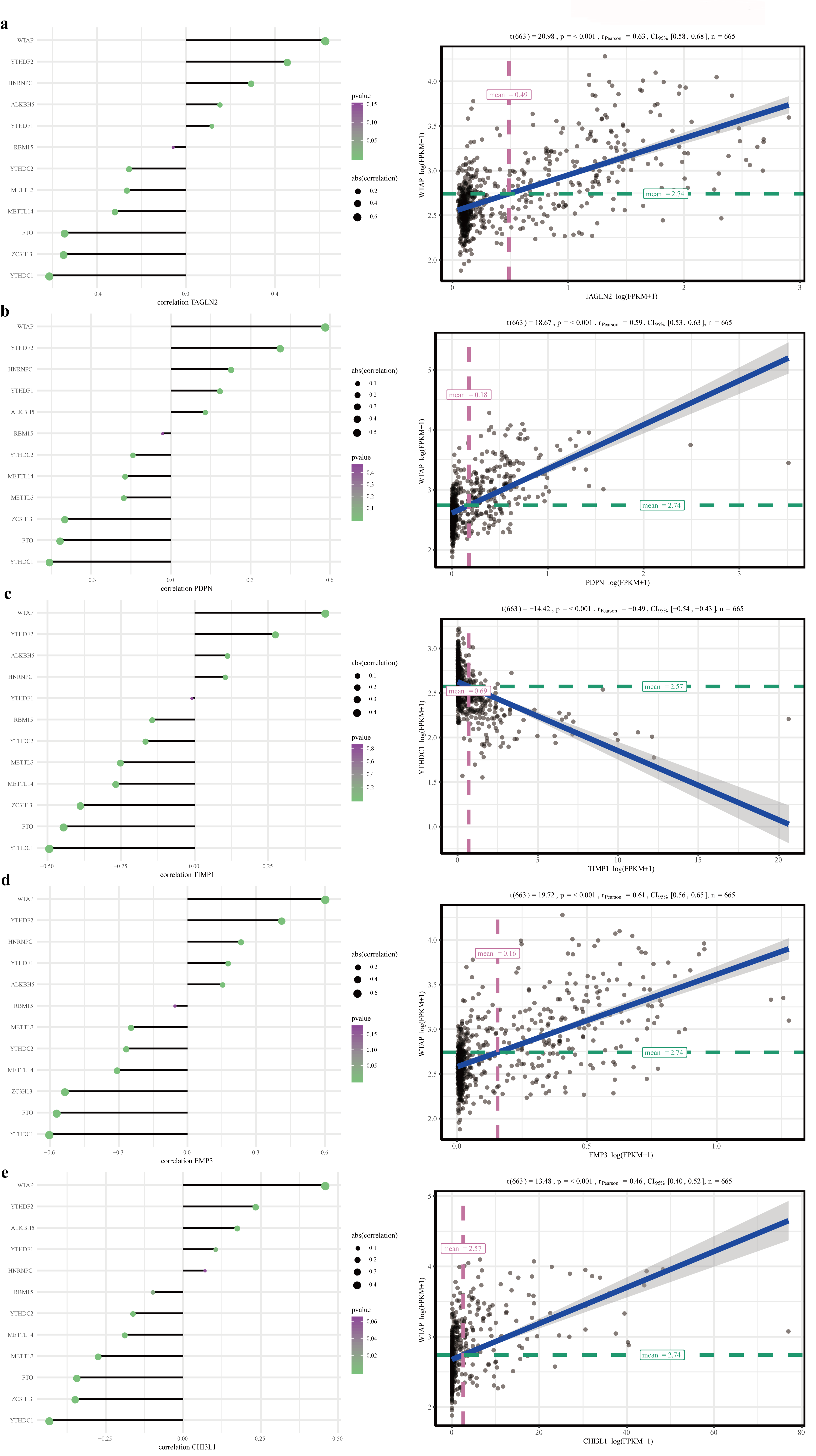

Supplement: Supplementary Figure 7 — Association of hub genes' expression with 12 m6A RNA methylation regulators in gliomas. (A) TAGLN2 (B) PDPN (C) TIMP1 (D) EMP3 (E) CHI3L1. [file Image_7.TIF]

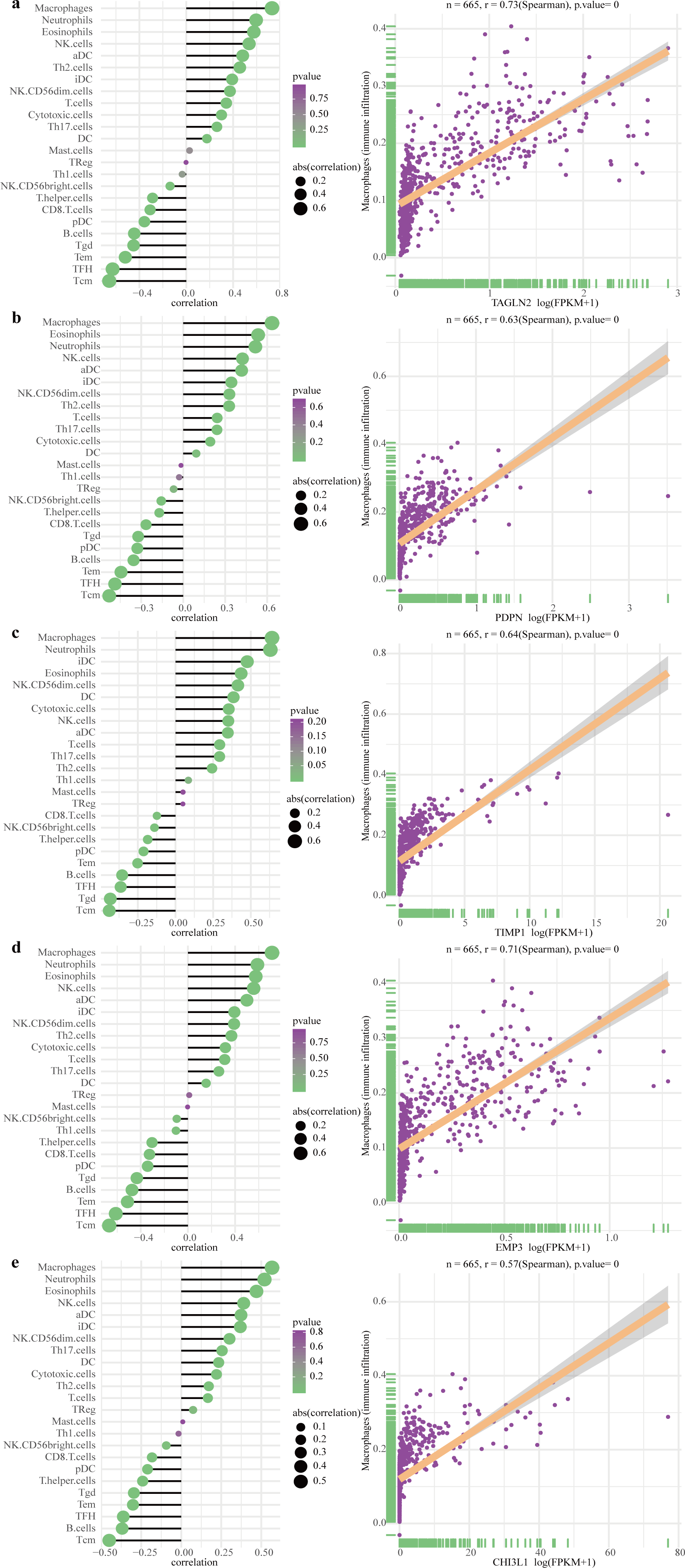

Supplement: Supplementary Figure 8 — Association of hub genes' expression with immune infiltration cells in gliomas. (A) TAGLN2 (B) PDPN (C) TIMP1 (D) EMP3 (E) CHI3L1. [file Image_8.TIF]

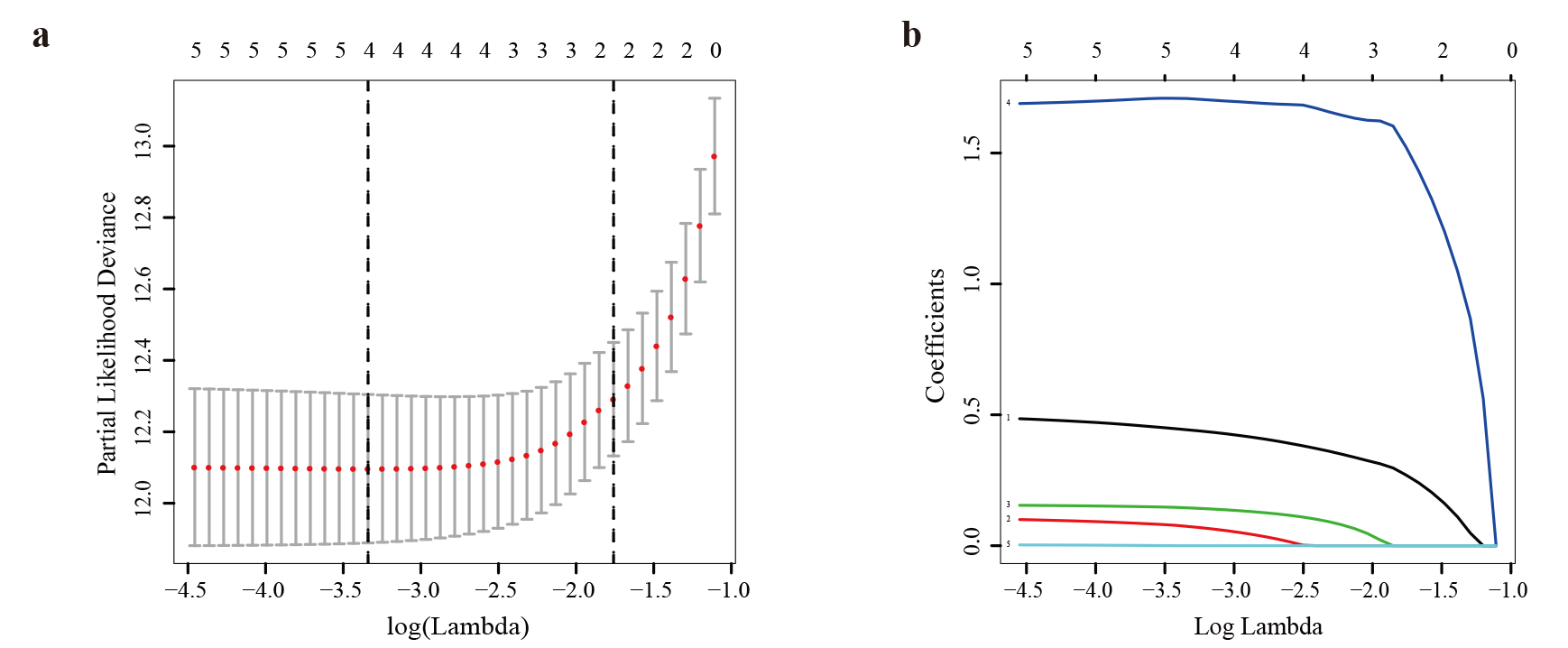

Supplement: Supplementary Figure 9 — (A,B) The process of building the risk scores containing 4 hub genes and the coefficients calculated by least absolute shrinkage and selection operator (LASSO) Cox regression algorithm are shown. [file Image_9.TIF]

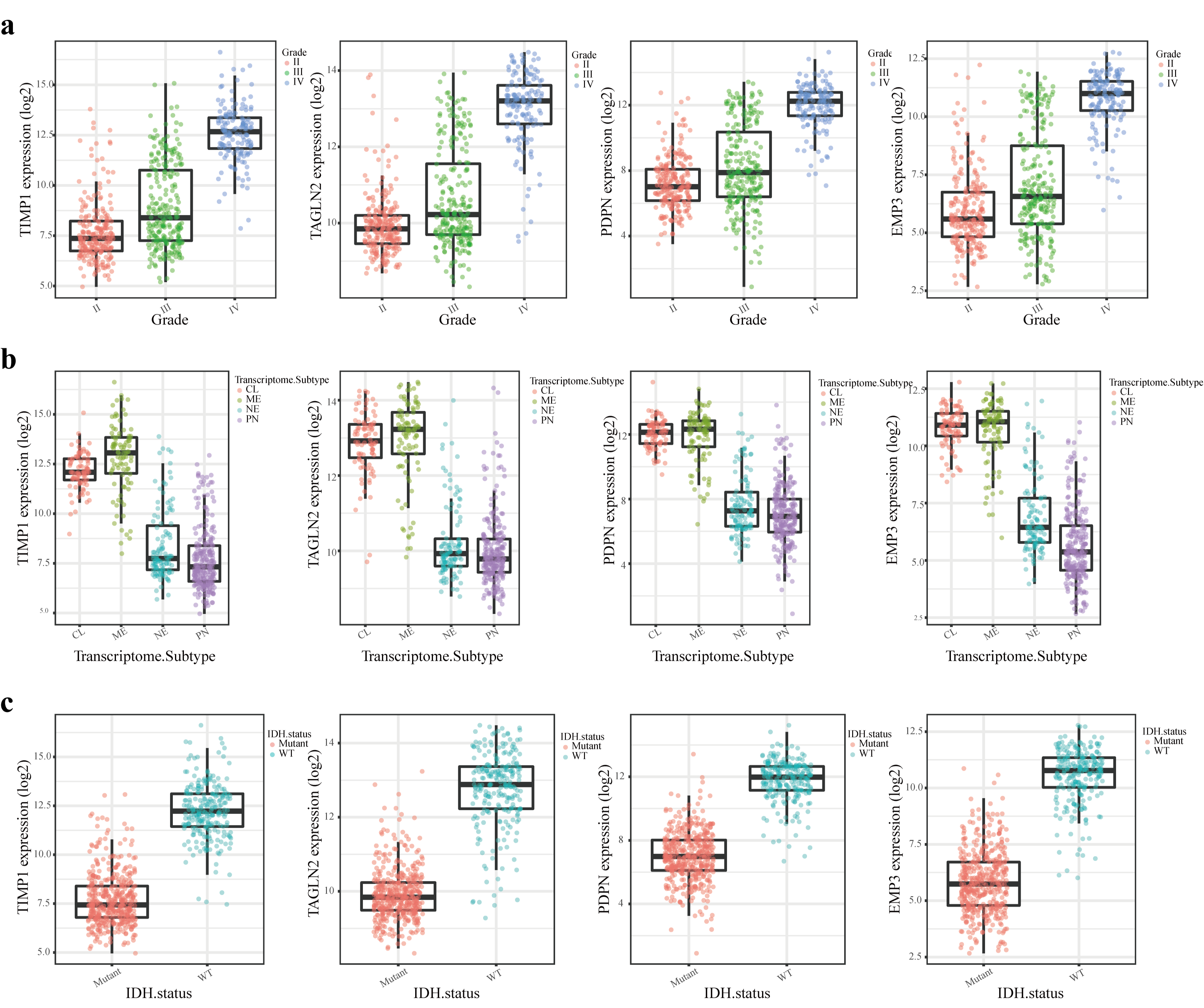

Supplement: Supplementary Figure 10 — Expression of 4 hub genes in gliomas with different clinicopathological features, from right to left, TIMP1, TAGLN2, PDPN, and EMP3, respectively. (A) The expression levels of 4 hub genes in gliomas with different WHO grades. (B) The expression levels of 4 hub genes in gliomas with different transcriptome subtypes. (C) The expression levels of 4 hub genes in gliomas with different IDH status. [file Image_10.TIF]

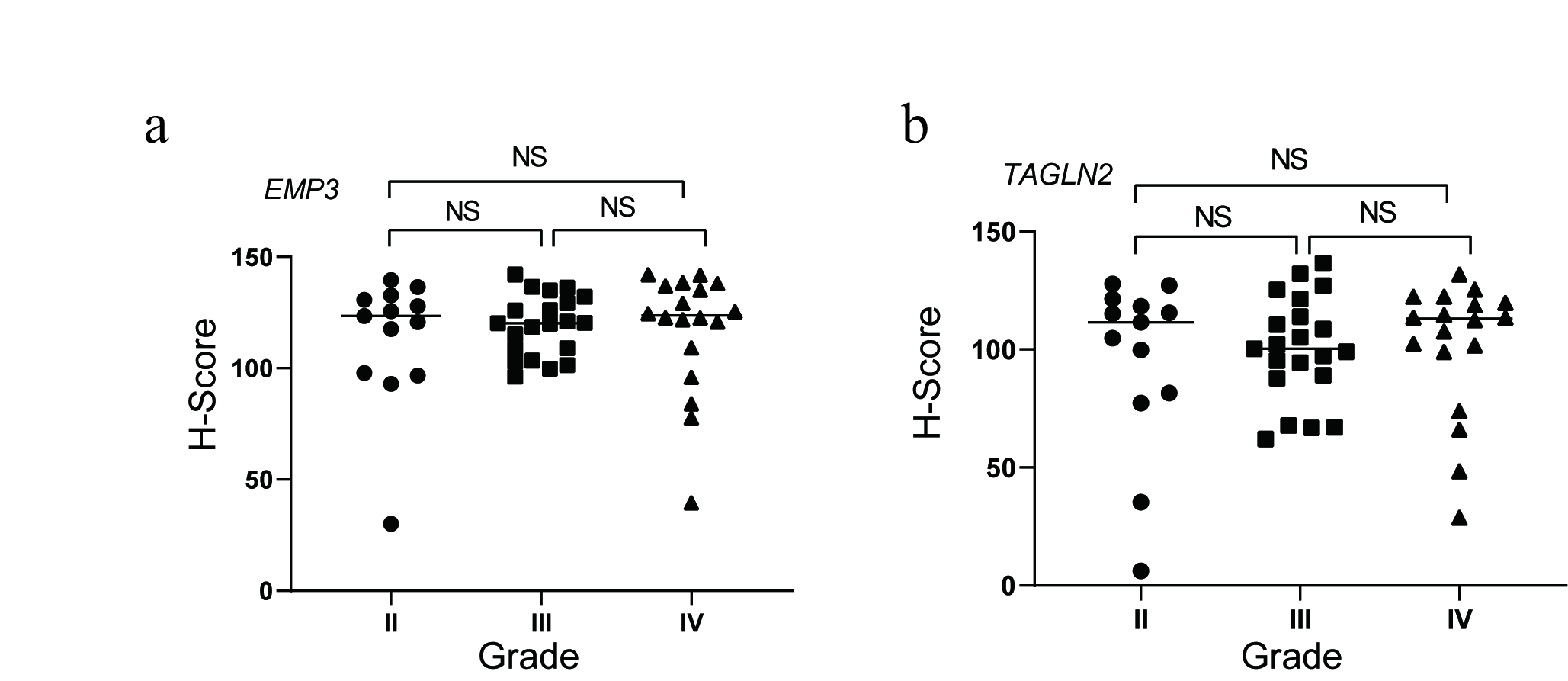

Supplement: Supplementary Figure 11 — H-score of EMP3 (A) and TAGLN2 (B) of glioma tissue-microarrays. [file Image_11.TIF]

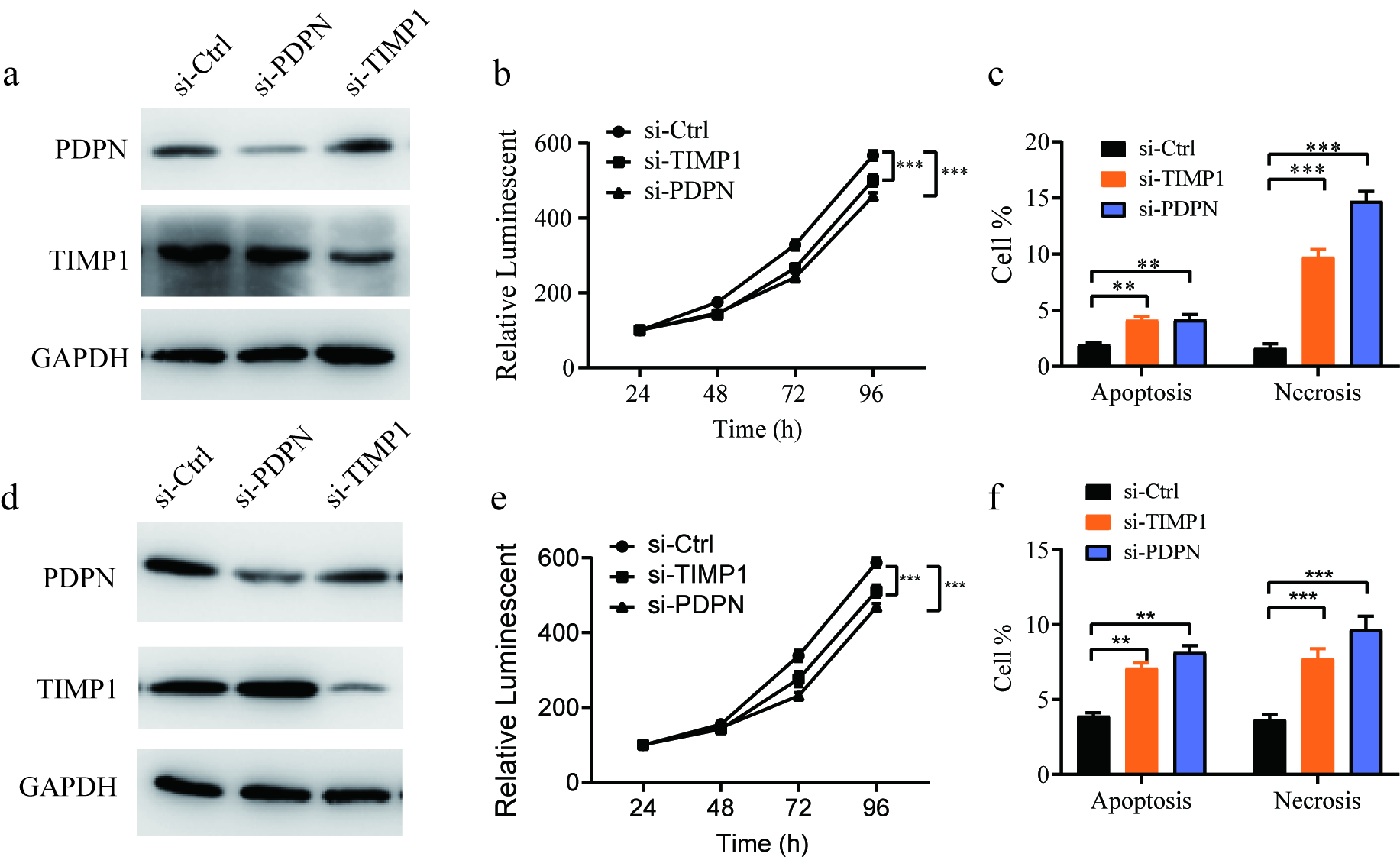

Supplement: Supplementary Figure 12 — (A,D) Western blot analysis validated the knockdown of TIPM1 or PDPN in U87 and A172 cells. (B,E) Cell proliferation was determined by ATP assay. (C,F) Flow cytometric analysis of Annexin V/PI staining in U87 and A172 cells after transfection with 50 nmol/L siTIMP1 or siPDPN and siCtrl for 72 h. [file Image_12.TIF]
